# Improving bone mineral density reporting to patients with an illustration of personal fracture risk

**DOI:** 10.1186/s12911-014-0101-y

**Published:** 2014-11-25

**Authors:** Stephanie W Edmonds, Peter Cram, Xin Lu, Douglas W Roblin, Nicole C Wright, Kenneth G Saag, Samantha L Solimeo, on Behalf of the PAADRN Investigators

**Affiliations:** Division of General Medicine, University of Iowa Carver College of Medicine, Iowa City, IA USA; College of Nursing, University of Iowa, Iowa City, IA USA; Faculty of Medicine, University of Toronto, Toronto, ON Canada; University Health Network and Mount Sinai Hospital, Toronto, ON Canada; Kaiser Permanente Georgia, Atlanta, GA USA; School of Public Health, Georgia State University, Atlanta, GA USA; Department of Epidemiology, University of Alabama at Birmingham, Birmingham, AL USA; Division of Clinical Immunology and Rheumatology, University of Alabama at Birmingham, Birmingham, AL USA; Department of Veterans Affairs, Center for Comprehensive Access & Delivery Research and Evaluation (CADRE), Iowa City Veterans Affairs Health Care System, Iowa City, IA USA

**Keywords:** Osteoporosis, DXA Scan, Risk, Fracture, Bone, Patient education

## Abstract

**Background:**

To determine patients’ preferences for, and understanding of, FRAX® fracture risk conveyed through illustrations.

**Methods:**

Drawing on examples from published studies, four illustrations of fracture risk were designed and tested for patient preference, ease of understanding, and perceived risk. We enrolled a convenience sample of adults aged 50 and older at two medical clinics located in the Midwestern and Southern United States. In-person structured interviews were conducted to elicit patient ranking of preference, ease of understanding, and perceived risk for each illustration.

**Results:**

Most subjects (n = 142) were female (64%), Caucasian (76%) and college educated (78%). Of the four risk depictions, a plurality of participants (37%) listed a bar graph as most preferred. Subjects felt this illustration used the stoplight color system to display risk levels well and was the most “clear,” “clean,” and “easy to read”. The majority of subjects (52%) rated the pictogram as the most difficult to understand as this format does not allow people to quickly ascertain their individual risk category.

**Conclusions:**

Communicating risk to patients with illustrations can be done effectively with clearly designed illustrations responsive to patient preference.

**Trial Registration:**

ClinicalTrials.gov Identifier: NCT01507662

## Background

Osteoporosis [OP] is a common disease of the skeletal system associated with fragility fractures. OP fractures, particularly of the hip, have been associated with increased morbidity and mortality, and decreased quality of life [1]. Because of these factors and the increased likelihood of chronic pain and dependence, persons with or who are at risk of developing OP will likely want to know their risk of having a fracture. Health care providers can quickly calculate an individual patient’s 10-year probability of hip and other major osteoporotic fractures combined (hip, vertebrae, distal forearm and proximal humerus) by using the World Health Organization’s FRAX® fracture risk assessment tool (www.shef.ac.uk/FRAX/) [2]. This web-based calculator uses 11 factors (age, race, sex, body mass index, prior history of fracture, parental history of fracture, secondary diseases, steroid use, smoking and alcohol intake, and bone mineral density [BMD] as determined by a dual energy X-ray absorptiometry [DXA]) to calculate an individual’s personalized 10-year absolute fracture risk. Providers typically use this risk calculation to communicate with patients when considering initiation of medication therapy and lifestyle counseling in the areas of nutrition, exercise, and tobacco and alcohol use. Because OP is asymptomatic and DXA alone is insufficient to predict fracture risk, providing individualized risk is an important strategy for reducing fracture risk and related morbidity. Unfortunately, it is unclear whether patients understand their DXA results, are able to act on this vital information in an appropriate way, or that health care providers know how to convey DXA results and FRAX® in a way that patients understand [3-7].

According to the Health Belief Model [HBM], prevention and adherence behaviors rely on patients knowing and understanding that they are at risk for a disease [8]. The HBM theorizes that patients will take necessary actions if they believe they are at risk for poor outcomes and that these poor outcomes can be prevented (e.g., osteoporotic fractures) by taking a recommended action (e.g., pharmacotherapy, calcium, weight-bearing exercises). In other words, patients may choose not to employ fracture risk reduction behaviors if they do not believe they are at risk, if they are not prepared to take action, or if they do not perceive benefits from the action. Accordingly, the OP prevention literature has demonstrated that patients at risk for fractures do not take actions to reduce their risks in part because they do not recognize their at-risk status [4,7,9-17].

The communication of risk is more complex than simply providing patients with information about their condition, its treatment, and sequelae. Health care providers seeking to communicate risk to patients must take into account their patients’ health and numeracy skills. Numeracy is the ability one has understanding risk concepts and basic probability [18,19]. As is the case for literacy [20], average numeracy in the United States is low [21]. Even highly educated samples perform poorly on fairly simple probability questions [18]. The consequences of poor numeracy for health behavior are high in the case of OP. For example, a 20% chance of major osteoporotic fracture in 10 years represents a high risk of sustaining a fracture, but patients may not understand that 20% is a high risk when receiving this number verbally or in a letter from their doctor [22].

Combining written, numeric, and illustrated representations of risk can ameliorate the effects of lower numeracy on patient understanding [23-26]. Studies to maximize patient comprehension of risk have employed a variety of images including bar graphs, pie charts, “thermometers,” and icon arrays. However, none of these studies have shown a single approach to be the most preferred or comprehensible to patients. In a study on comprehension of breast cancer risk, patients preferred an icon array to a bar graph [25]. Ghosh et al. found that combining a bar graph with an icon array lead to better understanding for patients who inaccurately perceived themselves to initially be at high risk of breast cancer [26]. Hill et al. found when presented with absolute risk of heart attack in the next five years, most patients preferred a risk thermometer [24]. Despite these promising directions, the published literature clearly lacks consensus on which depiction patients most easily understand, prefer, and are most motivated by [27,28]. In the field of OP, we were able to identify only a single trial that evaluated the use a pictogram to display fracture risk. The pictogram displayed personal risk of fracture and absolute risk reduction with pharmacotherapy and was used in a decision aid to help patients understand the relative benefits of taking bisphosphonates. Patients who used the decision aid were twice as likely to correctly identify their 10-year fracture risk as patients who did not see the pictogram [29].

We studied the feasibility of developing an instrument combining text and illustration to convey fracture risk to patients in a way that they prefer and understand. We set out to test patients’ preferences for, ease of understanding, and perceived fracture susceptibility after presenting subjects with a series of alternative illustrations depicting individualized risk of major fracture derived from a FRAX® score. Our objectives were to identify: which depictions patients prefer and why; whether patients can correctly ascertain fracture risk from the images and not over or underestimate risk; and which depiction is most associated with the desired health behavior outcome of seeking follow-up care from the referring provider. This study informed the instrument development for our current randomized clinical trial, The Patient Activation After DXA Result Notification (PAADRN) study, which tests the efficacy of a direct-to-consumer mailed DXA reporting intervention to activate patients for appropriate follow up with their health care providers based on test results [30].

## Methods

### Illustration development

Our team of health care practitioners, health literacy, and health communication experts developed depictions of FRAX® results. First, we reviewed published decision-making and risk communication literature to gain insight into which types of depictions had been shown to be effective at visually communicating risk [24,26]. Next, we created illustrations relevant to OP and FRAX®. The FRAX® calculation tool provides two scores: hip fracture risk and major osteoporotic (hip, shoulder, clinical spine, or forearm) fracture risk. We selected major osteoporotic fracture for this study because it addresses all bones and provides the highest percent risk of the two calculations, which may have more of an impact on a general population with low health numeracy. Risk categories of “normal”, “moderate”, and “high” risk for fracture were layered onto these illustrations. These categories were determined using treatment guidelines from National Osteoporosis Foundation, where a risk ≥20% is considered “high” risk for fracture [31].

**Figure 1:**
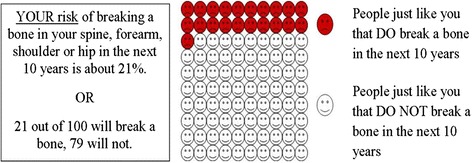
**Faces array.**

**Figure 2:**
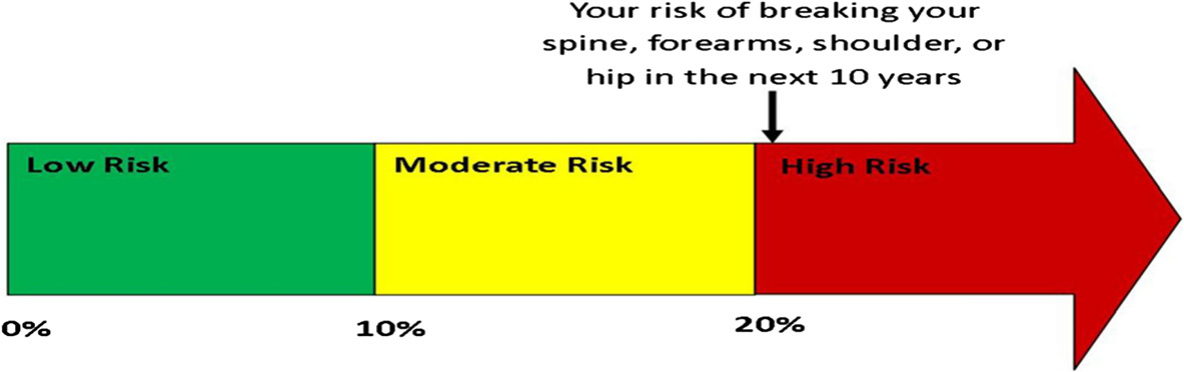
**Arrow.**

**Figure 3:**
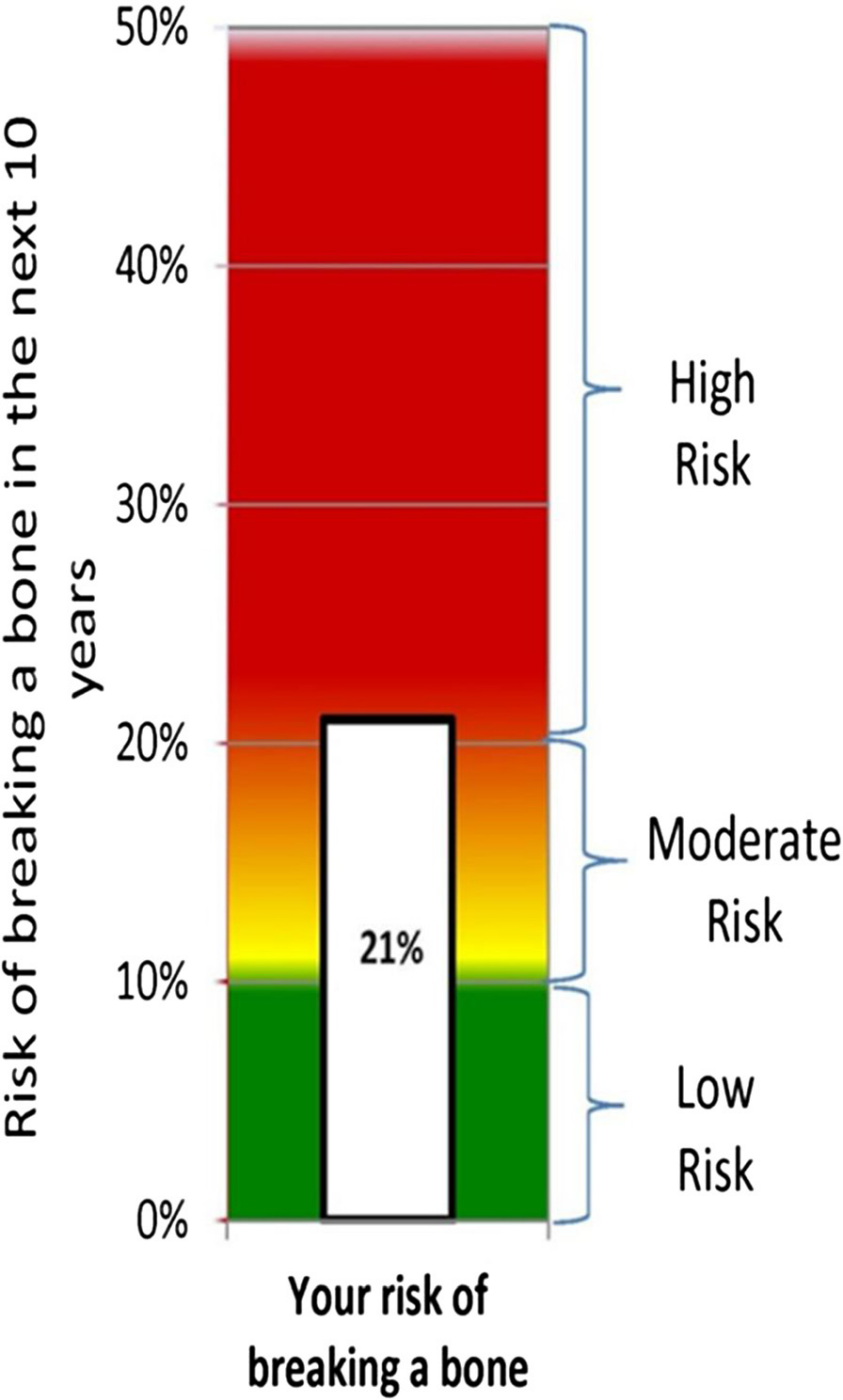
**Bar.**

We initially developed three illustrations, FACES, ARROW, and BAR, with each illustration depicting identical FRAX® results (i.e., a 10-year fracture risk of 21%) to conduct face validity evaluation. FACES (Figure 1) is a pictogram comprised of 100 faces. In our example, 79 were smiling and 21 were colored red and frowning, depicting a 21% risk. ARROW (Figure 2) is informed by the work of Hill, et al [24], and is a horizontally oriented arrow-shaped, directional graph that integrates a red, yellow, and green colored “stoplight” system to indicate risk: Low risk is associated with green, moderate is associated with yellow, and high risk is associated with red shading of equal widths. The BAR illustration (Figure 3), is informed by the work of Price et al., and employed a graduated stoplight color system, but is oriented vertically, similar to thermometer tools [32].

**Figure 4:**
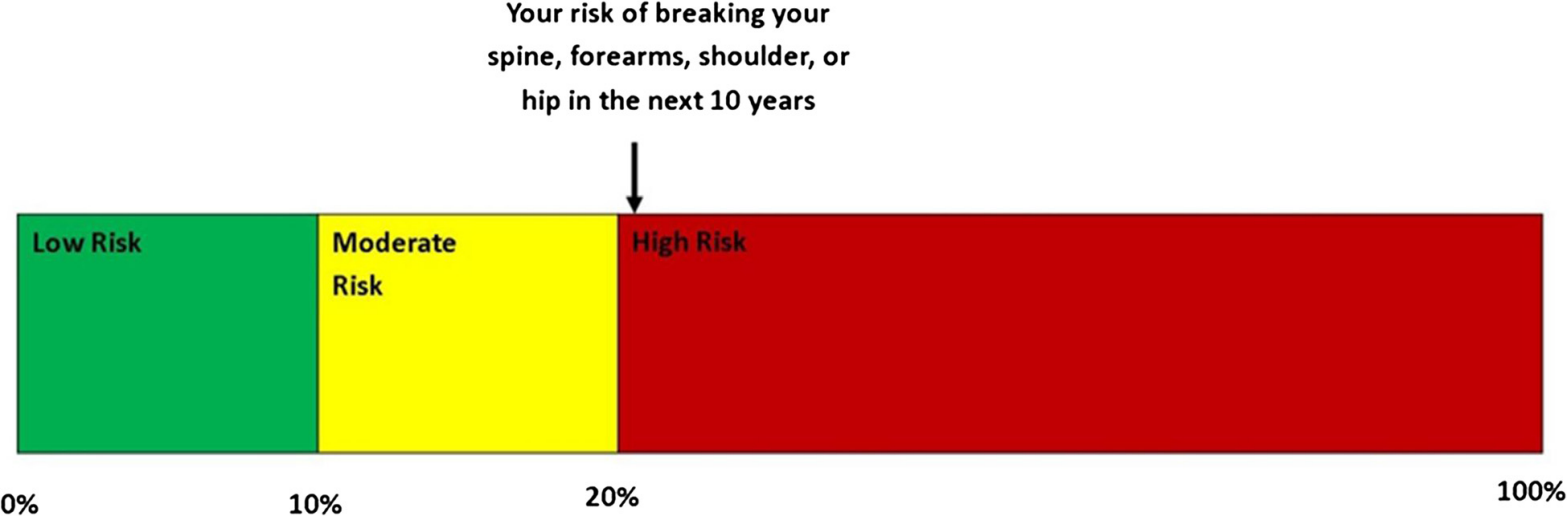
**Stoplight.**

Preliminary validity testing of these initial three illustrations was conducted with clinicians, health communication experts and a convenience sample of our target audience. Some members of our target audience disliked ARROW’s truncated scale so a fourth design, STOPLIGHT (Figure 4), was developed. As the name implies, the STOPLIGHT illustration integrates stoplight colors, but unlike the ARROW, STOPLIGHT is a rectangular depiction that does not imply progression and is scaled to 100% risk.

### Participants

For the more formal assessment, we recruited participants who were similar to our target audience for the PAADRN trial: English-speakers who were 50 years of age and older who had no visual or mental hearing impairments. We obtained our convenience sample (n = 142) from clinic reception and waiting areas at two sites, a large teaching hospital in the Midwestern United States (Site A) and a private clinic located in the southeastern part of the country (Site B). These two sites offer significant diversity in terms of geographic location and socioeconomic status of patients served (Site A is a rural safety net hospital while Site B serves primarily privately insured younger patients). We recruited participants by approaching them in-person (Site A) or by posting flyers in the clinic (Site B). Interviewers attempted to recruit a diverse sample of participants by approaching adults of varying ages, genders, and races and from a variety of clinic settings at each site. This study was approved by the Human Subjects Office at the University of Iowa and the Kaiser Permanente Georgia Institutional Review Board and due to the low anticipated risk to subjects, a waiver of written informed consent was granted.

### Study design and measures

We tested the four illustrations for preference, comprehension and perceived susceptibility using a combination of qualitative and quantitative measures. Participant preferences, comprehension, and perceived susceptibility were assessed using a structured interview. We explained the study to subjects in this way: *“Some people need to have a test called a DXA done to figure out how strong their bones are. After the test is done the results are sent to their doctor. In addition to this we want to send the test results directly to patients in a letter that will be easy for them to understand. … Now I am going to ask you to look at 4 different pictures. We would like to include a picture to help people better understand this risk of breaking a bone”.* Subjects were shown identical DXA results and associated fracture risk (21% or high risk) using the four illustrations discussed above. To control for potential confounding associated with priming participants, we presented the illustrations in a random order.

For each depiction, we asked participants a series of paired questions to obtain their ranked preference, ease of understanding, perceived susceptibility and underlying rationale for these perceptions. See Table 1 for examples of the paired items. We used items from the Subjective Numeracy Scale to obtain an independent measure of numeric comprehension [33] and three health literacy screening questions to measure literacy level [34]. At the end of the interview, subjects provided information regarding their history of DXA, osteoporosis status, fracture, and demographic data such as their sex, year of birth, educational attainment, race, and employment status. Participants who completed the 30 minute interview received a voucher for complimentary parking or a gift card to compensate them for their time.

**Table 1:** **Interview Schedule**

	**Mode of questioning**		**Construct assessing**		
	**Close-ended (Quantitative) questions**	**Open-Ended (Qualitative) questions**	**Preference**	**Comprehension**	**Perceived susceptibility**
**For each figure individually, presented to participants in random order**		What is this picture trying to show you?		✓	
	What would you say is the risk or chance of this person breaking a bone in the next 10 years?			✓	✓
	(Very high, High, Moderate, Low, Very Low)				
		What do you like about this picture? Why?	✓		
		What do you not like about this picture?	✓		
**For all figures, in comparison to one another**	Rank the pictures from your most favorite to your least favorite explain your decision.	Explain your decision.	✓		
	Which picture was easiest for you to understand?	Why?		✓	
	Which picture was hardest for you to understand?	What could we do to make it better?		✓	
	If these pictures showed YOUR risk, which one would make you most worried about breaking a bone?				✓

### Analyses

We used a sequential mixed methods approach to gain an in-depth understanding of participants’ stated preferences, ease of understanding and their underlying rationale [35]. Quantitative analyses were prioritized and analyzed first to guide the focus of qualitative inquiry. Interview data were entered into a preformatted text document and imported using preprocessor functions of a qualitative data analysis software platform, MAXQDA (Version 10, 2011). Preprocessing automatically links interview items with subject response and facilitates subsequent linkage of qualitative and quantitative analyses. The close ended, quantitative variables were then exported from the qualitative platform into SAS (Version 9.2 Cary, NC) for statistical analysis.

### Quantitative analysis

We compared demographic (e.g., age, race, sex) and clinical characteristics (e.g., history of prior DXA scans, history of osteoporosis or prior fracture) of subjects from the two sites. We used two-sampled t-test for comparisons of continuous variables and the chi-squared test for categorical variables. To evaluate how participants ranked preference for the illustration, we first assigned number 1 to the illustration that was most preferred to 4 for the illustration that was least preferred. Then we used Friedman’s test to compare the ranks of the most favorite to the least favorite among the four illustrations. Next, we examined differences in ranking between each pair of illustrations by running separate Wilcoxon signed-rank tests and applied Bonferroni correction on the test results. Alternatively, we used Cochran-Mantel-Haenszel statistics to assess whether the most preferred depictions differed among selected subgroups (e.g., men versus women, more versus less educated).

To examine the comprehension and perceived susceptibility of each picture, we first defined perception of risk by an answer to the question “What would you say is the risk or chance of this person breaking a bone in the next 10 years?” An answer of “High” risk was “correct”, other answers (e.g., “Very Low’, “Low’, “Moderate”, “Very High”) were “incorrect”. We deemed an answer of “High” as correct based on treatment guidelines from National Osteoporosis Foundation, where a risk ≥20% is considered “high” risk for fracture [31]. After that we compared the pictures’ ability to convey “correct” risk to subjects using logistic regression. We adjusted for average numeracy and accounted for the within-subject variance by treating subjects as random effect. Alpha level of 0.05 is considered statistically significant.

### Qualitative analysis

Two mechanisms were employed to maintain internal validity and reliability of the qualitative data analyses. First, while we employed a sequential mixed methods framework, initial coding of the open ended interview items was conducted concurrently and independently of quantitative analyses. This protected the qualitative investigators from inadvertently “discovering” the quantitative trends. Secondly, the qualitative investigators independently coded the open-ended interview items using a multi-coder team. In sum, data were imported into the qualitative software, automatically coded by topic using the preprocessor function of the program, and then subsequently coded for content by the lead qualitative coder. After the initial coding processes had been performed, the quantitative data were exported for analysis and the qualitative data team worked as a group to review and categorize subject rationales into themes. The themes were reviewed for face validity by the group and the data recoded by the team using the new codebook. Once the quantitative analyses had been finalized, the qualitative team reviewed data pertaining to perceived ease of understanding, preference, dislike, and areas for improvement in order to contextualize and understand the ranking results.

## Results

### Characteristics of participants

We interviewed 142 participants, a majority of whom were female (64%) and Caucasian (76%) (Table 2). Demographic characteristics differed between the two sites. Overall, Site B had a larger proportion of female (p-value = 0.03), African American (p-value <0.001), and highly educated (p-value <0.01) participants than Site A. Across sites more than half of subjects reported their health being “very good” or “excellent” (55%).

**Table 2:** **Characteristics of the study sample (n = 142)**

**Characteristics**	**All sites**	**Site A Sample 56%**	**Site B Sample 44%**	
Gender, %				
Female	64	56	74	*0.03
Age, %				
50-59	35	29	44	
60-69	39	41	36	
70+	26	30	21	
Education, %				
High school or less	22	30	13	*0.002
Some college	34	38	30	
College gaduate or more	44	32	57	
Race,%				
White	76	93	55	*< 0.001
Black	19	4	39	
Other	5	4	7	
Literacy, Mean (SD) Range 1-5 with higher number better literacy	3.3 (0.6)	3.4 (0.6)	3.2 (0.5)	
Numeracy, Mean (SD) Range 1-6 with higher number better numeracy	3.5 (1.4)	4.3 (1.2)	2.5 (0.9)	*< 0.001
General health, %				
Excellent	14	13	15	
Very good	41	46	36	
Good	37	34	40	
Fair	8	7	8	
Poor	1	0	2	
Bone health, %				
History of previous DXA	46	38	55	
History of osteoporosis or osteopenia	24	19	31	
Fracture history	16	13	19	

*Note:* *indicates variables for which the sites are significantly different (p < .05).

### Preference

When we examined participants’ ranking, there was a statistically significant difference in preference among the four illustrations (P < 0.001). Median interquartile range (IQR) of the ranks for ARROW, BAR, FACES and STOPLIGHT were 3 (2 to 3), 2 (1 to 3), 4 (2 to 4) and 2 (2 to 3), respectively. Bonferroni correction yielded a significance level set at p <0.008. There were no significant differences between ARROW and BAR (p = 0.03), ARROW and STOPLIGHT (p = 0.01), ARROW and FACES (p = 0.02), BAR and STOPLIGHT (p = 0.82). However, the overall ranking for FACES was significantly worse than BAR (p < 0.001) and STOPLIGHT (p < 0.001). Although ARROW, BAR, and STOPLIGHT appeared to be statistically similar, we chose BAR to be the winning illustration based on its median (IQR) statistic.

When we assessed the depiction that was ranked most preferred, a significantly higher proportion of respondents chose BAR as their most preferred illustration (selected by 37%) as compared to STOPLIGHT (selected by 24%), FACES (selected by 22%), or ARROW (selected by 17%) (p < 0.05), though FACES was ranked the least favorite by 65 subjects (52%). This coincided well with the findings using the entire ranking information. The participants ranked BAR as the most preferred by all age groups (33%, 31%, 39%), females (33%), males (36%), those that attended college (45%, 32%) and Whites (35%). FACES was ranked most preferred by two subgroups; non-Whites (Blacks 44% and other 40%) and those that did not attend college (34%). However, there were no significant differences for illustration preference among subgroups by age, race, sex, site, education attainment, or average numeracy Table 3.

**Table 3:** **Subjects’ preference for illustration format**

	**Favorite illustration (percentage who picked as their most preferred)**					
	**Arrow**	**Bar**	**Faces**	**Stoplight**	**No. missing**	**P-value**
Total (n = 127)	17	37	22	24	15	0.01
Gender, %						
Female (n = 81)	20	35	25	21	15	
Male (n = 46)	13	41	17	28		
Education, %						
High school or less (n = 27)	11	22	30	37	19	
Some college (n = 43)	21	47	12	21		
College graduate or more (n = 53)	17	38	25	21		
Age, %						
50-59 (n = 49)	18	37	18	27	15	
60-69 (n = 48)	21	33	21	25		
70+ (n = 30)	10	43	30	17		
Site,%						
Site A (n = 66)	18	38	15	29		
Site B (n = 61)	16	36	30	18	15	
Race, %						
White (n = 94)	18	38	17	27	16	
Black (n = 27)	19	33	37	11		
Other (n = 5)	0	20	40	40		

The primary reasons why respondents selected BAR as their favorite included the association of red color with the risk presented and the categorization of risk (low, moderate, and high) in association with the numeric value of reported risk. These positive appraisals are reflected in the following subject responses:

*“The color shows me I’m in high risk, without the color I would assume 21% is low”.*

*“I like how [BAR] is broken out with the colors and then that is reiterated with the scales of low, moderate, and high. And you got your percentages. And the colors really drive that all home”.*

*“[BAR] shows percentages of low, middle, high well and is easy to understand”.*

Research assistants then asked subjects why they ranked the other illustrations lower. One reason given was that the increasing risk implied by ARROW and that STOPLIGHT made it difficult to identify where one stood in regard to the overall risk represented by STOPLIGHT. FACES was reported least preferred because of its lack of clearly defined risk groups, the perceived “childlike” and “unprofessional” feel of FACES, and the disconnect between the risk reported and the perceived risk, as evidenced in these comments:

*“[FACES] doesn’t tell you what risk group you are in”.*

*“I feel like a three year old. And it makes me feel like my percentage isn’t high”.*

*“[FACES]: It is too confusing and too much going on. You want to see the numbers and not have to count them up”.*

### Ease of understanding, comprehension and perceived susceptibility

As expected, the illustrations participants preferred were also the ones reported as most easily understood. More subjects listed BAR (34%) than the other three illustrations. STOPLIGHT and ARROW were ranked second, listed as easiest to understand by 19% of participants. Participant characteristics were not significantly associated with reported difficulty in understanding at the 0.05 level of significance Table 4.

**Table 4:** **Subjects’ opinion on comprehension of illustration formats**

	**Easy to understand picture (percentage who picked as the easiest to understand)**					
	**Arrow**	**Bar**	**Faces**	**Stoplight**	**No. missing**	**P-value**
Total (n = 124)	19	34	27	19	18	0.06
Gender, %						
Female (n = 79)	22	33	32	14	18	
Male (n = 45)	16	36	20	29		
Education, %						
High school or less (n = 26)	19	19	35	27	21	
Some college (n = 42)	17	45	21	17		
College graduate or more (n = 53)	21	32	28	19		
Age,%						
50-59 (n = 48)	23	22	21	23	18	
60-69 (n = 48)	19	31	31	19		
70+ (n = 28)	14	39	32	14		
Site, %						
Site A(n = 62)	23	32	21	24	18	
Site B (n = 62)	16	36	34	15		
Race, %						
White (n = 91)	20	35	22	23	19	
Black (n = 27)	19	30	44	7		
Other (n = 5)	20	20	40	20		

When asked why they selected a particular depiction as easiest to understand, participants listed stoplight color associations, the relationship of individual risk to the risk groups, and overall formatting as reasons. The stoplight color offset of the individual risk from the bar graph in BAR was noted as helpful, as this participant explained, “*The ways they did the ranking and highlighted the different areas. It also puts your test score in with the white so you can actually see the scale”.* As for why other formats were perceived as more difficult to comprehend, subjects commented:

*“Counting all the little [FACES] and you don’t get a scale”.*

*“[FACES] Gives no feeling of where I stand as far as my risk”.*

*“[STOPLIGHT] It seems like there is nothing on the left side in the low risk, and you don’t have far to go to high risk”.*

*“When you look at [ARROW] and realize it’s not 100%, it’s deceptive”.*

In order to maximize the potential activation impact of the final illustration, for each depiction participants were asked ”What is this picture trying to show you?”, “What would you say is the risk or chance of this person breaking a bone in the next 10 years?”, and “Which picture would make you feel the most worried?” Across all illustrations, participants identified nine key messages. See Table 5. Participants’ comments about 10-year fracture risk were evenly represented among the illustrations, but comments about STOPLIGHT and ARROW more frequently mentioned that the image communicated “high risk”. In response to correctly matching the depiction to high risk, ARROW and STOPLIGHT had the highest correct risk perception (72% and 62% of responses, respectively). Different pictures were associated with recognizing the “correct” risk (p-value = 0.002). ARROW and STOPLIGHT had similar capacity to convey the risk correctly (odds ratio = 1.5, 95% CI = (0.9-2.6), p = 0.15) and were significantly better than BAR and FACE. For example, ARROW was 3.2 times better in rendering “correct” perception of risk comparing to BAR (95% CI = (1.9-5.5), p <0.001), and 37 times better than FACE (95% CI = (16.3, 84.9), p <0.001). Compared to STOPLIGHT, BAR is 4 times likely to represent “under” risk”. Compared to STOPLIGHT, FACES is 30 times likely to represent “under” risk. Most participants (75%) responded that FACES depicted a person with low risk of fracture, while 23% thought STOPLIGHT showed a very high risk. Lastly, a majority of participants thought BAR showed a moderate risk of fracture (58%). Average numeracy was not associated with recognizing the “correct” risk (p = 0.63).

**Table 5:** **Key risk messages identified by respondents**

**Risk message category**	**Associated responses**
**Fracture risk**	*“Risk of breaking a bone in spine, forearms, shoulders, or hip”.*
**High risk**	*“I’m in the high risk”.*
**Ten-year fracture risk**	*“In the next 10 years I am at a high risk for breaking those bones”.*
**General health risk**	*“Showing you from the high to low of risk involved”.*
**Personal fracture risk**	*“It is telling me where my bone density lies, and the risk of it”.*
**Preventable risk**	*“That you have 10 years to get your act together or you’ll be in the red”.*
**Action is required**	*“If you are in the red part you best be getting to the doctor”.*
**Bone density results**	*“Percent of risk of osteoporosis in spine, forearms, shoulder, or hip in the next 10 years”.*
**Risk categories**	*“It’s showing you are at moderate risk. High risk is clear up here. And it’s a little more than low risk*”.

Finally, participants rated STOPLIGHT AND ARROW as the most worrisome illustrations (33% and 34%, p <0.001). When asked why these illustrations instilled concern, typical responses included, “It [ARROW] points like it is going to get worse” and “[STOPLIGHT] would alarm you more because more length to the high risk and less to the moderate”.

## Discussion

In this mixed methods study, we found that participants preferred the BAR illustration and they did not prefer the icon array FACES. The use of stoplight colors to reflect fracture risk category was popular. We presented participants with four illustrations of identical risk in random order, asked them to rate their preference, ease of understanding, intent, and worrisome nature, and then asked them to explain their ratings. We found differences in participants’ response to the illustrations, demonstrating that the representation of risk has important bearing on patient’s consideration of health care information.

There was no strong consensus on the preferred illustration as none was preferred by more than half of participants. The BAR was most preferred of the four with 37% selecting it as their favorite option. Additionally, respondents listed BAR as the easiest to understand illustration. Our findings were similar to the findings of other studies that used a vertical bar graph to display risk [36,37]. McCaffery et al. found that participants had a strong preference for a bar graph versus an icon array, such as our FACES design. Additionally, they found that participants were more likely to accurately identify risks of >10% using a bar graph when compared to an icon array [36]. Hawley et al. also found that participants performed better on verbatim tasks (the ability to correctly read numbers from graphs) when shown a bar graph versus an icon array [38]. While participants reported that BAR was the easiest to understand of the four illustrations, they did not perform as well when asked about risk severity. Due to the participant feedback, we felt that the color gradation caused this confusion and not the bar-type format.

Risk communication studies are increasingly employing icon arrays as a mechanism for addressing low numeracy among patients. However icons arrays were least likely to lead to a correct identification of risk category by participants. Participants were least likely to prefer the icon array FACES. The FACES option likely suffered from a lack of risk stratification from low to moderate and then to high risks, a design feature in the other types of graphics that was very much preferred by participants. Our data suggest that patients with lower educational attainment may be more likely to prefer icon arrays to other illustrations; however, the difference between educational subgroups was not statistically significant. Additionally, we found that preference for a depiction was not significantly associated with average numeracy levels. Our finding that icon arrays are least preferred by some respondents may be in part due to the arrangement of negative icons within the array. There is evidence to suggest that random arrangement of icons within the array more effectively produces increased susceptibility [39], however open-ended comments by our respondents revealed that they found the depiction of a happy or sad face to be infantile, so it is unclear whether random arrangement would have surmounted that appraisal.

Use of stoplight colors emerged as a clear factor in respondents’ preferences and interpretation of information. The universal stoplight coloring system which equates the color green with health and positive movement, yellow with caution and slowed movement, and red with danger, was clearly internalized and applied to the interpretation of risk. Similar uses of the traffic light color system were used in other studies and found to be well perceived by participants in conveying risk [24,32]. However, the use of graduated colors along these shades in the BAR illustration was perceived to be confusing by respondents as evidenced by 44% of participants stating BAR depicted 21% risk of fracture as a moderate risk rather than as a high risk of fracture. While a color gradation was used in a prior study, there was no mention of their participants’ opinions of color gradation [32]. We conclude that, because the color graduated from orange to red slightly above the 20% line, participants still considered 21% to be moderate risk. Our stoplight coloring may limit a color-blind patient’s understanding of the depictions. While we used the color system to draw attention and aid in comprehension, we also provided labels to the categories so a color-blind individual could comprehend their risk level. Given the low prevalence of this disorder, specifically in women [40] who represent 80% of patients undergoing DXA, we feel that using this coloring system would be mostly beneficial.

Our study is not without limitations. Instead of running four separate experiments, we opted to test depictions sequentially in random order. Exposing all subjects to all illustrations may have primed them with greater reinforcement of the information when answering the questions for later illustrations; however we moderated this potential effect by randomizing the order in which illustrations were tested. Similarly, we tested three depictions that were somewhat different from one another and one (FACES) that was different from the others, rather than four similar illustrations. This purposeful variation was a methodologic approach to evaluate which depiction is preferred and led subjects to identify clinically correct risk level comprehension. For example, an icon array such as FACES can help patients visualize their true probability of fracture, but it does not aid them in understanding what is *clinically* considered high risk. This was evidenced by 75% of participants who considered a 21% as a low risk of fracture when viewing FACES. Our study sample was drawn from a general clinic population, which included by chance, some people who had knowledge of OP but others who had no prior knowledge of OP. Because some patients may have more knowledge of OP than others, we do not necessarily know if our results might differ between these two subgroups had samples been recruited specifically along these lines. However, for the purposes of PAADRN, this is not necessarily a limitation as we will be recruiting a similar mix of patients. For the purposes of the PAADRN study we wanted to learn which array was preferred and understood by the majority of participants in our target audience, patients undergoing DXA either for the first time or a repeat DXA and who are 50 years of age and older [30]. We found that non-Whites preferred FACES to other illustrations but this was not statistically significant. A larger sample of respondents with lower educational attainment and numeracy may have different responses. Additionally, we used a convenience sampling method which makes it difficult to ascertain that our quantitative results are reflective of the clinic population. However, we did use a more purposeful sampling technique to ensure that our sample was geographically, racially, and economically diverse. One illustration clearly emerged as the most preferred and most easily understood risk depiction among our target audience; however we concur with other researchers that a single risk illustration might not be unanimously accepted by all people [32].

To our knowledge, this is the first study to examine patient preference for communicating FRAX®. We found that numeracy was not significantly associated with preference, perceived ease of understanding, or correct identification of risk category for any of the illustrations examined in this study. Participants were asked to both rate their preferred format and to explain their reason for doing so, and this type of integration of qualitative and quantitative measures moves our understanding or risk communication forward. While participant preference was important in creating the depiction used for the PAADRN trial, we felt it was most important to use an illustration that patients both liked and which was associated with correct appraisal of fracture risk. Additionally, our goal in creating this depiction was not to recommend treatment but rather to choose a depiction that might motivate patients to communicate with their health care provider about their fracture risk or to make health behavior changes like increasing weight-bearing exercise or dietary calcium. The PAADRN trial will assess patient motivation and behaviors to improve their bone health upon receiving their illustration of personal fracture risk [30]. We found that the majority of participants in our sample significantly preferred and understood a bar-type graph to display 10-year fracture risk. This illustration could easily be added to DXA reports to aid health care providers and patients in making bone health care decisions. This type of work provides valuable and needed information to health care providers who want to improve DXA follow- up care by using illustrations to communicate personalized fracture risk using a depiction that is well perceived by a general audience of 50+ years.

## Conclusions

This study describes the methods used to develop a visual depiction of fracture risk that will be mailed with DXA results to patients in the intervention arm of the randomized clinical PAADRN trial. We found that participants had a significant preference for a vertically oriented risk depiction that provided clinically significant risk categories using a stoplight coloring system. As highlighted above, numeracy is an important consideration when communicating risk information to patients. Providing patients with a visual depiction of their personal risk of a disease or disease consequence may assist them in their understanding. However, careful thought should be taken when describing risk to patients, as many adults have low numeracy skills. When determining the best representation of risk obtaining feedback for preference and comprehension from a sample of patients in a target patient population is a critical first step in providing patients with an effective and acceptable risk depiction.

## Abbreviations

OP: Osteoporosis
BMD: Bone mineral density
DXA: Dual energy X-ray absorptiometry
HBM: Health belief model
PAADRN: Patient Activation After DXA Result Notification
